# Development of real-time PCR methods for quality control detection of pathogenic bacteria in cosmetic preparations

**DOI:** 10.3389/fpubh.2025.1572201

**Published:** 2025-04-24

**Authors:** Veronica Bolzon, Michela Bulfoni, Alessandro Nencioni, Emanuele Nencioni

**Affiliations:** ^1^Biofarma Group Srl., Udine, Italy; ^2^Department of Medicine, University of Udine, Udine, Italy; ^3^IBSA Institut Biochimique SA, Lugano, Switzerland

**Keywords:** real-time PCR (rt-PCR), cosmetics, quality control, method verification, ISO standards, pathogenic bacteria, DNA

## Abstract

**Introduction:**

The preservation of microbial safety in cosmetic products is essential for consumer health and requires rapid and accurate detection strategies… Traditional detection methods, such as quantitative and qualitative tests, are effective but often time-consuming and labor-intensive. Moreover, plate count methods fail to detect viable but non-cultivable cells, which remain alive but cannot grow under standard laboratory conditions. To address these limitations, molecular techniques like PCR, particularly real-time PCR (rt-PCR), multiplex rt-PCR, and viability PCR assays, as well as flow cytometry, have enhanced microbiological analysis by improving detection sensitivity, accuracy, and enabling rapid pathogen identification. ISO standards offer guidelines for reliable and consistent microbial detection methods, to guarantee the effectiveness of traditional and molecular techniques in food and cosmetic safety testing.

**Methods:**

This study evaluates real-time PCR (rt-PCR) as an alternative to the traditional plate-based method for the detection of *Escherichia coli, Staphylococcus aureus, Pseudomonas aeruginosa,* and *Candida albicans* in cosmetic formulations.

**Results and discussion:**

rt-PCR consistently demonstrated superior sensitivity and reliability, particularly in detecting pathogens at low inoculum levels and within complex matrices. For all pathogens, rt-PCR achieved a 100% detection rate across all replicates, reaching the same or superior results than the classical plate method. rt-PCR’s ability to directly target DNA overcomes issues related to colony morphology and microbial competition. The study highlights the necessity of standardized rt-PCR protocols aligned with international ISO guidelines to enhance its applicability in routine quality control programs. In conclusion, rt-PCR represents a significant advancement in microbial safety for food and cosmetics, offering a rapid, sensitive, and reliable alternative to conventional methods. By integrating enrichment strategies, rt-PCR ensures higher accuracy in pathogen detection, reinforcing product safety and regulatory compliance in the cosmetics industry.

## 1 Introduction

The development of rapid and reliable methods for the detection of microorganisms is fundamental to ensure consumer safety (1, 2). Manufacturers have to ensure that raw materials are free from contamination, production processes are secure, and the final product remains uncontaminated. This approach minimizes potential risks to health and enhances product quality (3).

Pathogenic bacteria can be classified into conventional pathogens, which infect hosts with normal immune defenses, and opportunistic pathogens, which typically affect individuals with compromised immunity. Among the pathogenic microorganisms that may contaminate food and cosmetics, only a small fraction is considered as pathogenic, capable of causing illness in consumers. Some examples include *Escherichia coli*, *Staphylococcus aureus*, *Listeria monocytogenes*, and *Pseudomonas aeruginosa*. For fungi, a specie of particular concern is *Candida albicans* (1, 3, 4).

Traditional detection approaches often involve plate count methods where samples are cultured on agar plates and incubated in order to count colonies. These methods are cost-effective, convenient and adaptable, but time-consuming, labor-intensive and may detect only viable cells (3, 5, 6).

Furthermore, the gold standard culture-based methods are operator-dependent, offer lower sensitivity, and the phenotypic properties on which the identification of bacteria is based can be equivocal, making the interpretation of the results difficult. Trained and highly qualified personnel are also required to carry out these investigations. Another limitation of traditional tests is their inability to detect the presence of viable, but non-cultivable cells, a very common physiological state (6–8).

To overcome these limitations, molecular technologies, such as nucleic acid amplification (PCR), have significantly improved the specificity and sensitivity of routine tests, reducing the time required for the detection of microbial pathogens (2, 3, 8). Among these methods, real-time PCR (rt-PCR) allow a real-time monitoring of DNA amplification through fluorescent signals proportional to the initial DNA template quantity. This method could serve as a rapid screening method for the detection of pathogens (3, 8, 9).

To ensure standardization in compliance with international norms, such as ISO guidelines, the development and implementation of rt-PCR protocols involve the 1. evaluation of sample preparation techniques tailored to the specific matrix to optimize DNA recovery and minimize interference; 2. assessment of method performance, including sensitivity, specificity, accuracy, and limit of detection; 3. comparison with standard reference methods to confirm reproducibility and consistency across replicates and 4. preparation of detailed protocols aligned international standards to ensure method reliability and acceptance in regulatory and industrial settings (1, 3, 8, 10).

Despite its advantages, rt-PCR integration into routine quality control is hindered by the lack of standardized protocols and variability in reagents (3). The development of molecular protocols requires to be entirely validated based on the available ISO guidelines, from the enrichment methods, the DNA extraction from matrices, and the PCR process itself.

All these challenges can be addressed by following to ISO-aligned methodologies, ensuring consistent and reliable pathogen detection for food and environmental safety (11–15). The International Organization for Standardization (ISO) standard for PCR-based detection of foodborne pathogens (16, 17) served as the foundational reference throughout this process.

The objective of our study was to evaluate the performance of a real-time PCR method for identifying major pathogens *Escherichia coli, Staphylococcus aureus, Pseudomonas aeruginosa,* and *Candida albicans* in different cosmetic formulations as a quality control measure in production. The method verification was conducted in accordance with ISO guidelines, and the results were compared with the gold standard culture method on agar plates. This work serves as a model demonstrating how to optimize an analytical molecular biology procedure for implementation in current microbiological techniques.

## 2 Materials and methods

### 2.1 Inoculation and enrichment procedures for cosmetics

Six commercial cosmetic products with varying ingredient compositions and physical characteristics (paste, compact solid, oily, creamy, milky) were selected (Table 1). Each was numbered 1–6 for simplified analysis.

**Table 1:** Cosmetic matrices involved in the study.

Reference number	Cosmetic type	Physical characteristics
1	Face cream	Creamy texture
2	Gel	Paste texture
3	Scrub	Oily texture with saline particles
4	Sun milk	Milky texture
5	Tanning oil	Oily texture
6*	Soap	Solid-state compact texture

*For this specific matrix, a 1:100 dilution is required.

Samples were spiked with low levels (3–5 CFU) of four common cosmetic pathogens—*E. coli, S. aureus, P. aeruginosa*, and *C. albicans*—with a blank sample included.

*S. aureus* was not tested in Matrix 6 due to antimicrobial ingredients (Caprylyl Glycol, Ethylhexylglycerine) (17). Reference strains (10–100 CFU/100 μL) were used for inoculation (Table 2).

**Table 2:** Reference quantitative strains used for the contamination of cosmetic matrices.

Reference strain	Reference	Batch	Expiration
ez accu shot *Candida albicans* derived from ATCC 10231	0443C	443–1,532-1	31/07/2025
ez accu shot *Escherichia coli* derived from ATCC 8739	0483C	483–1,352-3	31/07/2025
ez accu shot *Pseudomonas aeruginosa* derived from ATCC 9027	0484C	484–1,661-3	31/08/2025
ez accu shot *Staphylococcus aureus* derived from ATCC 6538	0485C	485–1,218-3	31/08/2025

Seven 1 g replicates of each cosmetic were diluted in 9 mL of Eugon broth (Biolife) per ISO methods. A plate count on non-selective media (TSA for *E. coli, S. aureus, P. aeruginosa*; SDA for *C. albicans*) confirmed pathogen concentrations per UNI ISO 7218:2024. TSA plates were incubated at 34°C for 4 days, SDA at 24.5°C for 6 days.

After confirmation, 10 μL of *E. coli, P. aeruginosa, C. albicans* and 7 μL of *S. aureus* were inoculated into all samples (excluding blanks) under a LAF hood to reach 3–5 CFU/g. Spiked samples were incubated at 32.5°C for 20–24 h. For the most complex matrix (Matrix 6), a 36-h enrichment incubation and a 1:100 dilution of the initial sample were required to detect positive samples for all pathogens. Additionally, a sample of each cosmetic without inoculation was incubated as a blank.

### 2.2 The gold standard analysis method defined by ISO

After 24 h of incubation, the enrichments were spread onto tripartite plates (*MCK/BPM/CET-Biolife, ref. 491,070*) for the detection of *Escherichia coli*, *Staphylococcus aureus*, and *Pseudomonas aeruginosa*, following ISO standards 21,150, 22,718, and 22,717, respectively. For the detection of *Candida albicans*, the same enrichments were spread onto SDA/CAF plates (*Biolife, ref. 4,020,062*), in accordance with ISO 18416. All plates were incubated at 32.5°C (range: 29–34°C), first for 24 h and then for an additional 24 h.

### 2.3 Automatic DNA extraction

DNA extraction was performed after the shortest enrichment time required by the ISO methods: 20 h.

Fungal and bacterial DNA was extracted from the enrichments of the contaminated samples and blanks. DNA was isolated using the PowerSoil Pro kit (*Qiagen GmbH, Hilden, Germany, ref. 47,014*) following the manufacturer’s instructions and processed with a QIAcube Connect extractor.

Prior to initiating the automated kit protocol, 250 μL of enrichments were mixed with 800 μL of CD1 solution, transferred into the PowerBead Pro Tube provided by the kit, and vortexed on a Vortex Adapter for 10 min at maximum speed. Lysates were centrifuged at 15,000 × g for 1 min, and 650 μL of supernatant was transferred to the Rotor Adapters. The adapters were then loaded onto the QIAcube Connect extractor as per the protocol.

Similarly, three extraction controls were processed: the medium control, zero control, and extraction control.

The extraction and elution volumes were selected according to the manufacturer’s instructions.

### 2.4 rt-PCR pathogen assays

For each pathogen, a rt-PCR plate was prepared, and each DNA extract was analyzed in duplicate.

Commercial rt-PCR kits, validated by the suppliers and including an internal reaction control, were used to analyze the extracts. For *Escherichia coli*, *Staphylococcus aureus*, and *Pseudomonas aeruginosa*, the R-Biopharm SureFast PLUS real-time PCR kit was used, while *Candida albicans* was analyzed with the Biopremier *Candida albicans* dtec-rt-PCR kit.

A rt-PCR plate was set up, and the thermal protocol was configured according to the suppliers’ instructions. Two reaction controls were included for each run: a no-template control (NTC) and a positive control provided in the kit.

For *Escherichia coli*, *Staphylococcus aureus*, and *Pseudomonas aeruginosa*, the following thermal conditions were applied: initial denaturation at 95°C for 1 min, followed by 40 cycles of 95°C for 10 s and annealing at 60°C for 15 s. Amplification was performed in a 25 μL final reaction volume, consisting of 19.3 μL of Reaction Mix, 0.7 μL of Taq Polymerase, and 5 μL of target DNA, using a QIAquant 96 instrument.

For *Candida albicans*, the thermal protocol included an initial activation at 95°C for 2 min, followed by 40 cycles of denaturation at 95°C for 5 s and hybridization/extension at 60°C for 20 s. Amplification was carried out in a 20 μL final reaction volume, consisting of 9 μL of DNase/RNase-free water, 5 μL of GPS-Mix, 1 μL of TargetSpecies dtec-rt-PCR-Mix, and 5 μL of target DNA, using the QIAquant 96 instrument.

All tests were conducted in duplicate.

## 3 Results

### 3.1 Inoculum level verification

According to ISO 7218, a plate count of 3–5 CFU is considered too low. To achieve a representative number of colonies for counting, a spread of 10 times the inoculum volume was performed.

Two plates with the inoculum volume and two plates with 10 times the inoculum volume were enumerated to verify the inoculum level.

Plate counts were carried out while ensuring proportionality between the different dilutions tested. To calculate the average number of colonies for the *n* and *n + 1* dilutions, the following equation was applied:

$$N=\frac{\sum c}{V\times \left[n_{1}+\left(0.1\times n_{2}\right)\right]\times d}$$

∑C = sum of the average numbers obtained from the count of the colonies on the plates in the 2 consecutive dilutions considered; V = volume (mL) of the inoculated amount in each plate; d = dilution factor corresponding to the first dilution considered, *n* = first dilution considered.

The inoculation levels per trial portion and the plate count results are expressed as CFU per trial portion and are presented in Table 3.

**Table 3:** Results obtained from plate count method of cosmetic samples inoculum.

	Inoculation (CFU)		10X inoculation (CFU)		Result (CFU/10 μL)	Result (CFU/trial portion)
	Plate 1	Plate 2	Plate 1	Plate 2		
*E. coli*	5	4	44	49	4,6	4,6
*S. aureus*	10	3	70	61	6,6	4,6
*P. aeruginosa*	3	3	27	29	2,8	2,8
*C. albicans*	4	5	35	42	3,9	3,9

### 3.2 Escherichia coli

Results from the rt-PCR analysis of the extracts for the detection of *Escherichia coli* were as expected and consistent with plate count results (Table 4). All samples and controls (100%) showed amplification in the VIC channel, which serves as the internal reaction control. In the FAM channel, corresponding to the target, all samples and their replicates (100%) amplified with valid Ct values. Additionally, the positive control and the extraction control were confirmed positive for the presence of *Escherichia coli*.

**Table 4:** Plate method and rt-PCR results obtained for the detection of *Escherichia coli*.

ISO method											rt-PCR method									
	Replicate											Replicate								
Cosmetic sample	1	2	3	4	5	6	7	Blank	Pos/neg		Cosmetic sample	1	2	3	4	5	6	7	Blank	Pos/neg
1	+	+	+	+	+	+	+	−	7/7	**=**	1	+	+	+	+	+	+	+	−	7/7
2	+	+	+	+	+	+	+	−	7/7	**=**	2	+	+	+	+	+	+	+	−	7/7
3	+	+	+	+	+	+	+	−	7/7	**=**	3	+	+	+	+	+	+	+	−	7/7
4	+	+	+	+	+	+	+	−	7/7	**=**	4	+	+	+	+	+	+	+	−	7/7
5	+	+	+	+	+	+	+	−	7/7	**=**	5	+	+	+	+	+	+	+	−	7/7
6*	+	+	−	+	+	+	+	−	6/7	**<**	6*	+	+	+	+	+	+	+	−	7/7

* an initial dilution 1to100 was applied.

The medium control, zero control, NTC, and all replicates of the blank sample were correctly negative.

### 3.3 Staphylococcus aureus

For the detection of *Staphylococcus aureus* in cosmetic samples, rt-PCR analysis provided superior results compared to the ISO method (Table 5). All samples and controls (100%) showed amplification in the VIC channel, serving as the internal reaction control. In the FAM channel, corresponding to the target, at least 6 out of 7 replicates for each cosmetic sample amplified with valid Ct values. With low inoculum levels, rt-PCR results were consistent with expectations. Plate results were satisfactory for most matrices but significantly underestimated for the creamy cosmetic (Matrix 1).

**Table 5:** Plate method and rt-PCR results obtained for the detection of *Staphylococcus aureus*.

ISO method											rt-PCR method									
	Replicate											Replicate								
Cosmetic sample	1	2	3	4	5	6	7	Blank	Pos/neg		Cosmetic sample	1	2	3	4	5	6	7	Blank	Pos/neg
1	−	−	−	−	−	−	−	**−**	0/7	**<**	1	+	+	−	+	+	+	+	**−**	6/7
2	+	+	+	+	+	+	−	**−**	6/7	**=**	2	+	+	+	+	+	+	−	**−**	6/7
3	+	+	+	+	+	+	+	**−**	7/7	**=**	3	+	+	+	+	+	+	+	**−**	7/7
4	−	+	+	+	+	+	+	**−**	6/7	**<**	4	+	+	+	+	+	+	+	**−**	7/7
5	+	+	+	+	+	+	+	**−**	7/7	**=**	5	+	+	+	+	+	+	+	**−**	7/7

For cosmetic sample n=6 the test is not applicable.

Additionally, the positive control and the extraction control were confirmed positive for the presence of *S. aureus*. The medium control, zero control, NTC, and all replicates of the blank sample were correctly negative (see Table 5).

### 3.4 Pseudomonas aeruginosa

For the detection of *Pseudomonas aeruginosa* in cosmetic enrichments, no significant differences were observed between rt-PCR results and those obtained using the ISO method. All samples and controls (100%) showed amplification in the VIC channel, confirming the functionality of the internal reaction control. In the FAM channel, specific to the target, at least 5 out of 7 replicates of each cosmetic sample with a low inoculum level amplified with valid Ct values. Additionally, the positive control and extraction control were confirmed positive for the presence of *Pseudomonas aeruginosa*.

The medium control, zero control, NTC, and all replicates of the blank sample were correctly negative (see Table 6).

**Table 6:** Plate method and rt-PCR results obtained for the detection of *Pseudomonas aeruginosa*.

ISO method											rt-PCR method									
	Replicate											Replicate								
Cosmetic sample	1	2	3	4	5	6	7	Blank	Pos/neg		Cosmetic sample	1	2	3	4	5	6	7	Blank	Pos/neg
1	+	+	−	+	−	+	+	−	5/7	=	1	+	+	−	+	−	+	+	−	5/7
2	+	−	+	+	−	−	+	−	4/7	**<**	2	+	−	+	+	+	−	+	−	5/7
3	+	+	+	+	+	+	+	−	7/7	=	3	+	+	+	+	+	+	+	−	7/7
4	+	+	+	+	−	+	+	−	6/7	=	4	+	+	+	+	−	+	+	−	6/7
5	+	+	+	+	+	+	+	−	7/7	=	5	+	+	+	+	+	+	+	−	7/7
6*	+	+	−	+	+	+	−	−	5/7	**<**	6*	+	+	−/+	+	+	+	−	−	6/7

* an initial dilution 1to100 was applied.

Replicates that tested negative by rt-PCR were also confirmed negative using the gold standard ISO method. This may be attributed to inoculum variability due to the small sample volume used.

### 3.5 Candida albicans

For the detection of *Candida albicans*, the enrichment time was extended to 36 h. The inoculum volume, verified by plate count, corresponded to 3.9 CFU/g. With a 36-h enrichment, rt-PCR demonstrated improved sensitivity, with at least 4 out of 7 replicates testing positive for *C. albicans* across all cosmetic formulations. In contrast, the ISO method showed limited sensitivity for samples with low cell levels. Specifically, *C. albicans* was not detected in any replicates of cosmetics 1 (creamy texture) and 2 (gel texture), and only 1 out of 7 replicates of cosmetic 6 (soap) tested positive for the pathogen.

To improve detection rates, a 1:10 dilution of low-level contaminated cosmetic samples, followed by a 36-h enrichment, resulted in more than 55% positivity for *C. albicans*. To achieve 100% positivity, an additional initial dilution (1:100) was performed. In this case, 2 out of 2 replicates of the more complex matrices 1, 2, and 6 tested positive after a 1:100 dilution and a 36-h enrichment (Table 7).

**Table 7:** Plate method and rt-PCR results obtained for the detection of *Candida albicans* after 36 h of enrichment.

ISO method (36 h-enrichment)											Rt-PCR method (36 h-enrichment)									
	Replicate											Replicate								
Cosmetic sample	1	2	3	4	5	6	7	Blank	Pos/neg		Cosmetic sample	1	2	3	4	5	6	7	Blank	Pos/neg
1	−	−	−	−	−	−	−	**−**	0/7	**<**	1	−	+	−	+	+	+	−	**−**	4/7
2	−	−	−	−	−	−	−	**−**	0/7	**<**	2	−	+	−	+	−	+	+	**−**	4/7
3	−	−	+	+	−	+	+	−	4/7	**<**	3	−	−	+	+	+	+	+	−	5/7
4	+	+	+	+	+	+	−	−	6/7	**<**	4	+	+	+	+	+	+	−	−	6/7
5	+	+	+	+	+	+	+	−	7/7	=	5	+	+	+	+	+	+	+	−	7/7
6*	−	+	−	−	−	−	−	−	1/7	**<**	6*	+	+	+	−	−/+	+	−	−	5/7

* an initial dilution 1to100 was applied.

## 4 Discussion

The application of rt-PCR demonstrated a clear advantage over the ISO plate-based methods for the detection of *Escherichia coli, Staphylococcus aureus, Pseudomonas aeruginosa*, and *Candida albicans* in cosmetic samples, also at of low inoculum levels and complex matrices. While the plate methods are well-established, showed limitations in sensitivity, especially for *C. albicans*, where nonspecific growth on SDA/CAF plates was observed in the presence of other pathogens (3, 4, 7).

For *E. coli, S. aureus,* and *P. aeruginosa*, rt-PCR provided reliable and consistent results compared to the gold standard, with amplification detected in all replicates under standard enrichment conditions. The detection of *C. albicans*, however, had required some protocol adjustments, including extending the enrichment time to 36 h and, for more complex matrices, applying an initial higher dilution (1:100). These modifications were fundamental for improving sensitivity, particularly in challenging formulations such as creamy, soap or gel textures. The 1:100 dilution, combined with a 36-h enrichment, resulted in 100% positivity in replicates for *C. albicans*, highlighting the importance of optimizing protocols for specific pathogens and sample types.

Furthermore, the variability observed in plate results, especially for low inoculum levels, underlines the potential limitations of traditional methods in detecting pathogens in complex cosmetic formulations. The ability of rt-PCR to detect target DNA directly and avoid issues of nonspecific growth makes the molecular test a superior alternative, where conventional methods struggled to deliver consistent results.

This study highlights the effectiveness of rt-PCR in detecting bacterial and fungal contaminants in cosmetic samples, offering significant advantages over ISO plate-based methods. The results confirm that rt-PCR is highly sensitive and reliable, particularly for pathogens present at low concentrations or in complex matrices (2, 8, 9).

Traditional plate-based methods may struggle to selectively and reliably detect pathogens in cosmetic formulations, particularly in the presence of competing microorganisms or complex sample compositions (6, 7, 18).

Although rt-PCR represents a powerful alternative to conventional microbiological methods, its integration into routine quality control workflows must be justified by a cost–benefit analysis. While the initial investment in specialized instrumentation and reagents for rt-PCR is considerably higher than that required for traditional plate-based methods, the reduction in analysis time, enhanced sensitivity, and automation potential often outweigh operational costs in high-throughput or time-sensitive environments. Moreover, faster decision-making enabled by molecular diagnostics can reduce production downtime and prevent distribution of contaminated products, further offsetting costs (2, 8, 17, 19).

However, rt-PCR presents certain limitations. A major issue is the potential for false-positive results caused by the high sensitivity and the possible the detection of residual DNA from non-viable microorganisms. Conversely, false negative results may arise from PCR inhibition due to matrix effects or suboptimal DNA extraction (19, 20). Another limitation of rt-PCR lies in its inability to discriminate between live and dead microorganisms, as the assay targets bacterial DNA. To address this, viability PCR approaches combining nucleic acid intercalating dyes such as propidium monoazide with rt-PCR have been developed to selectively exclude DNA from dead cells, enhancing result relevance in the context of microbial risk assessment (19, 20). This discrimination between viable and non-viable cells can also be achieved through flow cytometry, whereas even classical plate culture methods fail to distinguish between live and merely viable-but-non-cultivable cells (21–23). In this light, rt-PCR can be considered a valuable screening tool due to its high sensitivity, with positive results subsequently confirmed by the gold standard or an orthogonal method. Our findings suggest that rt-PCR, combined with tailored enrichment protocols, can serve as a robust tool for microbial quality control in the cosmetics industry, ensuring higher accuracy, reproducibility, and sensitivity compared to conventional methods.

## 5 Conclusion

This study demonstrates the clear advantages of rt-PCR over traditional ISO plate-based methods for detecting microbial contaminants in cosmetic samples. rt-PCR offers superior sensitivity, particularly in at low inoculum levels and in complex cosmetic matrices, where plate methods showed limitations, especially for *C. albicans.* The optimization of enrichment protocols is essential for maximizing detection accuracy. Given its reliability, reproducibility, and specificity, rt-PCR represents a powerful tool for microbial quality control campaigns in the cosmetics industry, ensuring enhanced safety and compliance with regulatory standards.

## Data Availability

The original contributions presented in the study are included in the article/supplementary material, further inquiries can be directed to the corresponding author.
